# Supplementary material for: Imagery of movements immediately following performance allows learning of motor skills that interfere
Source: Sci Rep. 2018 Sep 25;8:14330. doi: 10.1038/s41598-018-32606-9 (PMC6156339; doi:10.1038/s41598-018-32606-9)
Supplement: Supplementary file 1 — Supplemental Information [file 41598_2018_32606_MOESM1_ESM.pdf]

Supplementary Information:
Imagery of movements immediately following performance allows learning of motor skills that interfere

Hannah R. Sheahan*, James N. Ingram , Goda M. Žalalytė , Daniel M. Wolpert

Computational and Biological Learning Laboratory, Department of Engineering, University of Cambridge
*Corresponding author: sheahan.hannah@gmail.com

To encourage uniformity of movement kinematics, we placed constraints on the timing of participants' reaches (see Methods). If unperturbed movements to the central target were substantially different for the two possible secondary targets, this could have facilitated learning [4, 5]. Therefore, we examined whether the kinematics of pre-exposure movements to the central target within each group depended on which secondary target (±45°) was displayed.

We extracted position data from when the hand left the starting location until 50 ms after it entered the central target. We tested for baseline differences in peak speed, movement duration (which covaries with peak speed), maximum signed lateral deviation and path length. Preparatory neural activity has been shown to differ as a function of peak speed [1], hand path curvature [3], and movement extent [2, 6, 7], and such differential activity during planning might affect learning.

For each group and kinematic measure we performed a repeated-measures ANOVA on the pre-exposure null trials as a function of follow-through direction (±45°). Of the 20 tests (Table 1), we found one marginally significant difference (motor imagery path length, p=0.046), however the mean difference in path length between left and right secondary target positions was only 0.13cm, which other studies have shown is insufficient to drive learning [5].

Table S1. Movement kinematics on pre-exposure (null field) trials do not depend on secondary target position. Data are for the movement to the central target, and from a single start position (0°). δ is the difference between mean measures for each target (+45° target minus −45° target).

	Follow-through			Planning only			No motor imagery			Motor imagery			Motor imagery no fixation		
Measure	δ	$F_{1,7}$	p	δ	$F_{1,7}$	p	δ	$F_{1,15}$	p	δ	$F_{1,15}$	p	δ	$F_{1,7}$	p
Lateral deviation (cm)	-0.26	0.49	0.507	-0.37	0.85	0.387	-0.12	0.93	0.350	-0.27	2.96	0.106	-0.52	3.54	0.102
Path length (cm)	-0.01	0.00	0.953	-0.45	4.76	0.065	-0.01	0.02	0.901	-0.13	4.72	0.046	0.11	0.96	0.361
Duration (s)	-0.01	0.06	0.807	-0.03	0.56	0.478	-0.00	0.55	0.472	-0.01	1.06	0.319	0.01	0.14	0.724
Peak Speed (cm/s)	-0.8	0.69	0.434	-0.6	0.15	0.711	-1.0	2.16	0.163	-0.8	2.12	0.166	0.1	0.02	0.890

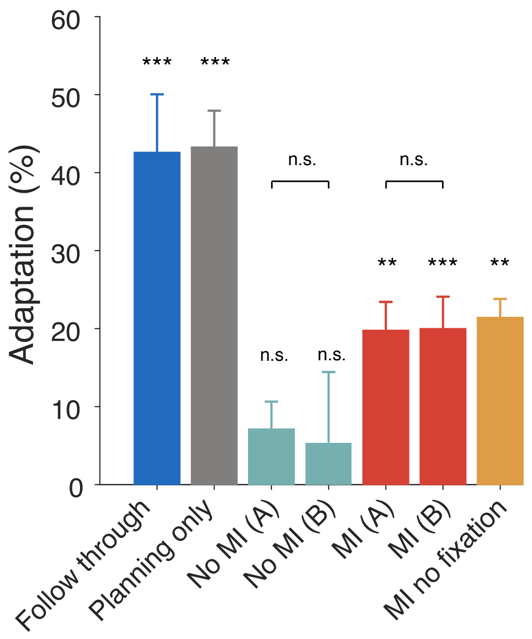

Figure S1. Final adaptation for all subgroups Final adaptation measured on follow through channel trials for all participants across the subgroups of the motor imagery group (MI (A) and MI (B)) and the no motor imagery group (No MI (A) and No MI (B)) (mean ± s.e. of final six blocks of exposure). The subgroups denoted by (A) performed the post-exposure phase immediately after the exposure phase, and the subgroups denoted by (B) performed an additional phase in which we included channels on the non-follow through trials. Asterisks show statistical significance of final adaptation level compared to pre-exposure. MI = motor imagery; n.s. = not significant.

References

[1] Mark M Churchland, Gopal Santhanam, and Krishna V Shenoy. Preparatory activity in premotor and motor cortex reflects the speed of the upcoming reach. *Journal of Neurophysiology*, 96(6):3130–3146, 2006.

[2] Q J Fu, J I Suarez, and T J Ebner. Neuronal specification of direction and distance during reaching movements in the superior precentral premotor area and primary motor cortex of monkeys. *Neurosurgery*, 70(5):2097 – 2166, 1993.

[3] S Hocherman and Steven P Wise. Effects of hand movement path on motor cortical activity. *Experimental Brain Research*, pages 285–302, 1991.

[4] Ian S. Howard, Daniel M. Wolpert, and D. W. Franklin. The effect of contextual cues on the encoding of motor memories. *Journal of Neurophysiology*, 109(10):2632–2644, 2013.

[5] Eun Jung Hwang, Opher Donchin, Maurice A. Smith, and Reza Shadmehr. A gain-field encoding of limb position and velocity in the internal model of arm dynamics. *PLoS Biology*, 1(2), 2003.

[6] Julie Messier and John F Kalaska. Covariation of primate dorsal premotor cell activity with direction and amplitude during a memorized-delay reaching task. *Journal of neurophysiology*, 84(1):152–165, 2000.

[7] A Riehle and J Requin. Monkey primary motor and premotor cortex: single-cell activity related to prior information about direction and extent of an intended movement. *Journal of neurophysiology*, 61(3):534–49, 1989.
